# The immune microenvironment in endometrial carcinoma: mechanisms and therapeutic targeting

**DOI:** 10.3389/fimmu.2025.1586315

**Published:** 2025-07-17

**Authors:** Yilin Wang, Nana Liu, Xiangcui Guo, Ruobing Han, Jin Bai, Jiateng Zhong, Qianqing Wang

**Affiliations:** 1 Department of Gynecology , Xinxiang Central Hospital, Xinxiang, China; 2 Xinxiang Medical University, Xinxiang, China; 3 The Fourth Clinical College, Xinxiang Medical University, Xinxiang, China; 4 Henan Medical Key Laboratory for Immunology and Targeted Therapy in Gynecological Oncology, Xinxiang, China; 5 Xuzhou Medical University, Xuzhou, China

**Keywords:** endometrial carcinoma, tumor immune microenvironment, immune evasion, treatment resistance, targeted therapy, immunotherapy

## Abstract

Endometrial carcinoma (EC) represents one of the most prevalent malignancies within the female reproductive system. The frequency of its occurrence is on the rise annually, and patients diagnosed at advanced stages face a less favorable prognosis. Recent studies have highlighted the significant influence of the tumor immune microenvironment (TME) on the initiation, progression, metastasis, and therapeutic resistance of endometrial cancer. The TME encompasses various components such as tumor-associated macrophages (TAMs), myeloid-derived suppressor cells (MDSCs), cancer-associated fibroblasts (CAFs), immune cells, and the extracellular matrix (ECM). These elements contribute to an immunosuppressive milieu by secreting cytokines, extracellular vesicles (EVs), and engaging immune checkpoint pathways like PD-1/PD-L1, thereby supporting tumor immune evasion and resistance to treatment. This review synthesizes current understanding of the EC-TME, focusing on the distinct roles and interactions of its key constituents within the context of EC biology. Furthermore, we explore the rationale and progress for novel therapeutic strategies targeting the TME, such as immune checkpoint inhibitors, combination therapies, and nano delivery systems leveraging EVs, aiming to provide insights for improving EC patient outcomes.

## 1 Introduction

Endometrial cancer (Endometrial Cancer, EC) is a common malignant tumor in gynecology. The global incidence and mortality rates of EC have been increasing persistently, and it has become the tumor with the highest incidence in the female reproductive system in some developed countries, especially with a high prevalence among postmenopausal individuals (1). Alarmingly, epidemiological shifts reveal a marked increase in incidence among younger women and specific ethnic groups, such as women under 50 in Puerto Rico, highlighting the heterogeneous nature of EC risk and distribution (2). The insidious onset of EC, often with non-specific or absent early symptoms, contributes to a high proportion of patients presenting with advanced-stage disease, which severely compromises therapeutic efficacy and prognosis (3).

The crucial challenges influencing the prognosis of EC are centered on the high recurrence rate (20 - 30%) and treatment resistance, which is particularly prominent in advanced cases (4). Recent research has disclosed that the dynamic regulation of the tumor microenvironment (Tumor Microenvironment, TME) plays a core role herein. The TME is composed of tumor cells, immune cells (such as TAMs, TILs), stromal components, and cytokines, etc., and drives tumor progression through multiple mechanisms: Immunosuppressive cells (M2 macrophages, Tregs) weaken the anti-tumor immune response; Remodeling of the extracellular matrix promotes invasion and metastasis; Factors such as TGF-β and TNF-α mediate immune escape and chemotherapy resistance (5, 6). Crucially, the unique composition and signaling within the EC-TME not only dictates disease aggressiveness but also unveils novel targets for therapeutic intervention, such as immune checkpoint blockade, offering hope for improving patient survival (4).

In recent years, as research on the Tumor Microenvironment (TME) has deepened continuously, targeted therapeutic strategies aimed at immune cells such as Tumor-Associated Macrophages (TAMs), Myeloid-Derived Suppressor Cells (MDSCs), and Cancer-Associated Fibroblasts (CAFs), along with stromal components, have gradually emerged as a research focus. For example, reshaping the immune microenvironment and enhancing the anti-tumor immune response are expected by reprogramming the polarization state of TAMs, inhibiting the functions of MDSCs, or regulating the activities of CAFs. Furthermore, extracellular vesicles (EVs), as crucial mediators of intercellular communication and by carrying bioactive molecules such as miRNAs and proteins, mediate immune suppression and chemotherapy resistance, thereby offering new therapeutic targets. Nevertheless, despite certain advancements in existing research, the complexity and heterogeneity of the TME still present substantial challenges for treatment.

This review comprehensively examines the current landscape of the EC-TME. We detail the critical cellular players – TAMs, MDSCs, and CAFs – and non-cellular components, particularly EVs, emphasizing their EC-specific functions and interplay. We then critically evaluate evolving therapeutic strategies designed to target these elements, including the integration of single-cell sequencing insights to understand TME heterogeneity. Finally, we discuss future research directions and clinical trial design considerations, aiming to provide a foundation for advancing precision immunotherapy in endometrial cancer.

## 2 The components and dynamic interactions of the tumor microenvironment in endometrial cancer

### 2.1 Immune cell clusters

In the tumor immune microenvironment (TME) of endometrial cancer, immune cell populations play a pivotal role. These cells modulate tumor growth, invasion, and immune evasion through the secretion of cytokines, chemokines, and metabolic products. The following sections detail the principal immune cells and their functions:

#### 2.1.1 Tumor-associated macrophages

TAMs constitute one of the most abundant immune cell types within the TME and are primarily classified into M1 (anti-tumor) and M2 (pro-tumor) phenotypes (7, 8). Within the EC-TME, M2-polarized TAMs typically predominate. These cells secrete immunosuppressive cytokines (e.g., IL-10, TGF-β), directly inhibiting CD8+ T cell activity and cytotoxic function (9–11). Concurrently, M2 TAMs produce potent pro-angiogenic factors like VEGF (12), fostering the development of new blood vessels that supply nutrients and oxygen essential for tumor growth and metastasis (13). Clinically, a high density of M2 TAMs within EC tumors is strongly correlated with increased tumor grade, stage, lymphovascular space invasion, and reduced overall survival, establishing them as key contributors to EC aggressiveness and poor prognosis (14). Consequently, strategies aimed at modulating TAM polarization (e.g., promoting M1 or repolarizing M2 to M1) represent a promising therapeutic approach to reinvigorate anti-tumor immunity in EC (15).

#### 2.1.2 Myeloid-derived suppressor cells

MDSCs (Myeloid-Derived Suppressor Cells) constitute a heterogeneous population of myeloid cells characterized by their immunosuppressive properties. This category includes mononuclear MDSCs (M-MDSCs) and polymorphonuclear MDSCs (PMN-MDSCs). MDSCs suppress T cell proliferation through the secretion of arginase and reactive oxygen species (ROS), thereby dampening the anti-tumor immune response (14, 16). The accumulation of MDSCs in the peripheral blood and tumor tissue of EC patients is significantly associated with advanced disease stage, metastatic spread, and inferior survival outcomes (17, 18). Studies specifically in EC have demonstrated that MDSC abundance disrupts the balance of tumor-infiltrating immune cells, creating an environment permissive for tumor progression and resistance to immunotherapy (19). Therefore, targeting MDSCs to alleviate immunosuppression is a rational strategy to enhance treatment efficacy in EC (20).

#### 2.1.3 Cancer-associated fibroblasts

Cancer-associated fibroblasts (CAFs) play a pivotal role in the tumor microenvironment, originating from diverse cell types including epithelial cells, endothelial cells, and mesenchymal stem cells (MSCs). The transformation of these cells into CAFs is mediated by various mechanisms. Epithelial cells undergo epithelial-mesenchymal transition (EMT), which is regulated by multiple signaling pathways within the tumor microenvironment (21). Endothelial cells, particularly during tumor angiogenesis, contribute to CAF formation by secreting cytokines and growth factors that promote CAF activation and function (22). MSCs can also be induced to differentiate into CAFs through signals within the tumor microenvironment, resulting in CAFs with potent immunosuppressive properties that facilitate tumor immune evasion (23).

**Figure 1:**
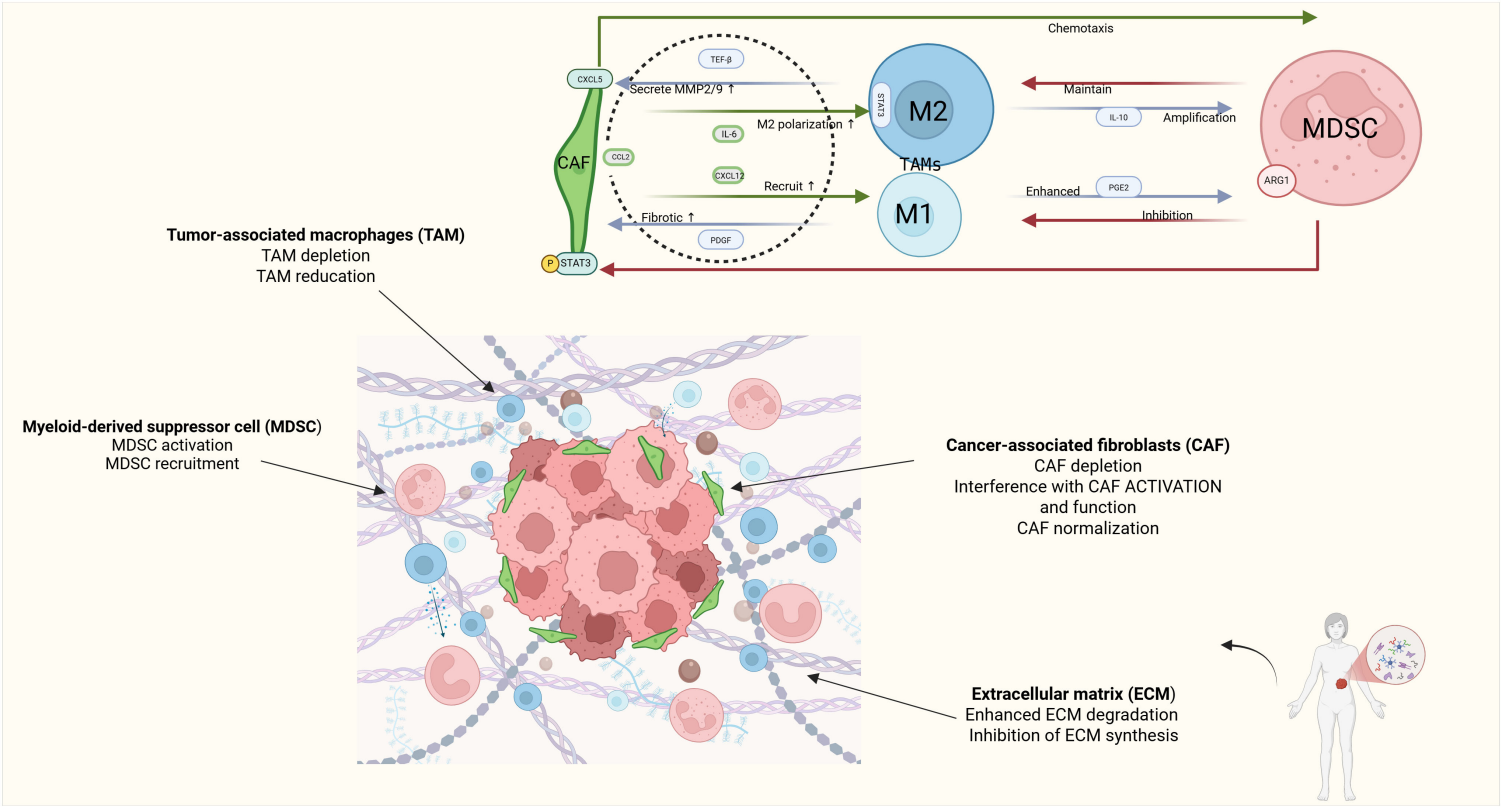
Schematic representation of key cellular interactions in the EC tumor microenvironment. Created in BioRender. yilin, w. (2025) https://BioRender.com/kgamfy8.

CAFs remodel the extracellular matrix (ECM) by secreting cytokines such as FGF and IL-6, as well as growth factors, thereby enhancing tumor invasiveness and metastatic potential (24, 25). In EC, CAFs secrete a plethora of growth factors (e.g., FGF, HGF) and cytokines (e.g., IL-6, TGF-β). These factors directly stimulate EC tumor cell proliferation and survival while also recruiting and activating immunosuppressive cells like TAMs and MDSCs, thereby reinforcing the immunosuppressive niche (26). Critically, CAFs in EC contribute significantly to chemoresistance. They secrete factors like glutathione (GSH) that protect tumor cells from oxidative stress induced by platinum-based drugs and can alter drug availability or metabolism (27). The activation state and specific markers of CAF subpopulations in EC are emerging as potential prognostic indicators and therapeutic targets (28) (**Figure 1**).

### 2.2 Extracellular vesicles and signaling networks in cellular communication

Extracellular vesicles (EVs) are small membrane-bound structures secreted by cells and are ubiquitously present in various body fluids, including blood, urine, and lymph. Based on their cellular origin and biological properties, EVs can be categorized into three primary types: exosomes, microvesicles, and apoptotic bodies (29). EVs play a crucial role in intercellular communication by transporting diverse bioactive molecules, such as miRNAs, proteins, and lipids, thereby modulating the function and behavior of recipient cells (30). Within the EC-TME, EVs play multifaceted and significant roles in driving tumor progression and therapy resistance (31, 32).

#### 2.2.1 EV cargo in EC pathogenesis

EVs derived from EC tumor cells and stromal cells carry specific molecules that directly influence tumor behavior. For instance, EVs can transport oncogenic miRNAs (e.g., miR-223) that target tumor suppressor genes or pathways involved in apoptosis, enhancing tumor cell survival and proliferation (33). They also carry proteins like P-glycoprotein (P-gp), a drug efflux pump, contributing directly to multidrug resistance (MDR) by reducing intracellular chemotherapeutic drug concentrations (34).

#### 2.2.2 EVs in immunosuppression

EVs are potent mediators of immune suppression within the EC-TME. They can deliver immunosuppressive ligands (e.g., PD-L1), cytokines (e.g., TGF-β, IL-10), and regulatory miRNAs directly to immune cells like T cells and NK cells, inhibiting their activation, proliferation, and cytotoxic functions (4). EV-mediated transfer of specific miRNAs can also reprogram immune cell phenotypes, for example, promoting the differentiation of monocytes into M2 macrophages (35, 36).

#### 2.2.3 EVs in angiogenesis and metastasis

EVs facilitate EC progression by promoting angiogenesis and invasion. They transport pro-angiogenic factors (e.g., VEGF, FGF) that stimulate endothelial cell proliferation and new vessel formation. Furthermore, EVs carry matrix-remodeling enzymes (e.g., MMPs, uPA) that degrade the basement membrane and ECM, clearing a path for tumor cell migration and invasion into surrounding tissues and vasculature (37). EVs also prepare distant metastatic niches by influencing the phenotype of cells at potential secondary sites.

The critical role of EVs in mediating communication between EC tumor cells and the various TME components positions them as attractive targets for novel diagnostics and therapeutics aimed at disrupting these pro-tumorigenic networks.

## 3 The dual roles of tumor-associated macrophages in endometrial cancer

TAMs are not merely passive bystanders but active participants in EC pathogenesis, exhibiting complex and context-dependent functions primarily dictated by their polarization state.

### 3.1 Phenotypic polarization and functionality

TAMs in EC display significant heterogeneity, existing along a spectrum between classically activated M1 (anti-tumor) and alternatively activated M2 (pro-tumor) phenotypes (3). M1 TAMs, typically induced by microbial products or Th1 cytokines (e.g., IFN-γ), secrete pro-inflammatory cytokines (TNF-α, IL-12, IL-1β), generate reactive oxygen and nitrogen species (ROS/RNS), and promote antigen presentation, thereby stimulating anti-tumor immune responses. In contrast, M2 TAMs, polarized by Th2 cytokines (e.g., IL-4, IL-13, IL-10), secrete immunosuppressive factors (IL-10, TGF-β, PGE2), express scavenger receptors, and produce pro-angiogenic factors (VEGF, FGF, PDGF), actively supporting tumor growth, angiogenesis, tissue remodeling, and immune evasion (38) Metabolic reprogramming underpins the functional divergence between M1 and M2 TAMs. M1 TAMs utilize glycolysis and the pentose phosphate pathway, converting arginine to nitric oxide (NO) via inducible nitric oxide synthase (iNOS), which has cytotoxic effects. M2 TAMs, however, rely on oxidative phosphorylation and fatty acid oxidation, metabolizing arginine to ornithine and polyamines via arginase-1 (ARG1), promoting cell proliferation and tissue repair (39). In the endometrial cancer microenvironment, lactic acid and hypoxic conditions are critical factors that induce the polarization of TAMs toward the M2 phenotype. Lactic acid inhibits mitochondrial function in TAMs, promoting their polarization to the M2 type and establishing a positive feedback loop of immunosuppression (40). This polarization process not only enhances the immunosuppressive capabilities of TAMs but also further weakens the anti-tumor immune response, ultimately facilitating tumor progression (41).

### 3.2 The mechanism of treatment resistance

The predominance of M2 TAMs within the EC-TME directly contributes to resistance against both chemotherapy and immunotherapy through several interconnected mechanisms (42):

#### 3.2.1 Immune checkpoint modulation

M2 TAMs frequently express high levels of immune checkpoint ligands, particularly PD-L1. Interaction of PD-L1 on TAMs with PD-1 on T cells delivers inhibitory signals that dampen T cell activation, proliferation, cytokine production, and cytotoxic capacity, effectively blunting anti-tumor immune responses (43, 44). The polarization state influences this; M2 TAMs generally exhibit higher PD-L1 expression and stronger immunosuppressive capacity via this pathway compared to M1 TAMs (45). TAMs located in hypoxic tumor regions or near blood vessels are often hotspots for PD-L1 expression and T cell suppression.

#### 3.2.2 Activation of pro-survival pathways

TAMs secrete a multitude of factors (e.g., EGF, FGF, IL-6) that activate key pro-survival and proliferative signaling pathways in EC tumor cells, such as PI3K/Akt/mTOR and JAK/STAT pathways (46). Activation of PI3K/Akt signaling promotes tumor cell survival, proliferation, and metabolism, while also conferring resistance to apoptosis induced by chemotherapeutic agents. TAM-derived factors can also activate the NF-κB pathway in tumor cells, promoting inflammation, cell survival, and inhibition of apoptosis (47, 48). Collectively, these signals enhance tumor cell resilience to therapy.

#### 3.2.3 Sustaining an immunosuppressive niche

Beyond direct effects on tumor cells and T cells, M2 TAMs contribute to a broader immunosuppressive environment by secreting cytokines (IL-10, TGF-β) that inhibit dendritic cell maturation, promote Treg differentiation and function, and recruit additional immunosuppressive cells like MDSCs. They also produce enzymes (e.g., IDO) that deplete tryptophan, further suppressing T cell function.

Targeting TAMs, particularly strategies to deplete M2 TAMs, block their recruitment, or reprogram them towards an M1 phenotype, is therefore a critical component of overcoming therapy resistance in EC.

## 4 The synergistic cooperation between MDSCs and CAFs

### 4.1 The immunosuppressive functionality of myeloid-derived suppressor cells

MDSCs are potent suppressors of anti-tumor immunity in EC, utilizing diverse mechanisms to inhibit effector immune cell function and promote a tolerant microenvironment (49):

#### 4.1.1 Cytokine-mediated tumor promotion and immune suppression

MDSCs secrete significant amounts of cytokines like IL-6 and TGF-β within the EC-TME. IL-6 acts as a potent growth factor for EC tumor cells, activating the JAK/STAT3 pathway, which promotes proliferation, survival, and invasion (50, 51). Furthermore, IL-6 contributes to immunosuppression by promoting the polarization of monocytes towards M2 TAMs and inhibiting dendritic cell function (52).

TGF-β is a master regulator with pleiotropic effects. It directly suppresses the activation and function of T cells, NK cells, and macrophages. Crucially, TGF-β is a potent inducer of epithelial-mesenchymal transition (EMT) in EC tumor cells, enhancing their invasive and metastatic potential. It also stimulates CAF activation and ECM production (53).

#### 4.1.2 Inhibition of lymphocyte function via soluble mediators

MDSCs employ enzymatic pathways to directly inhibit T and NK cell function. High expression of ARG1 depletes extracellular L-arginine, an essential amino acid for T cell function. L-arginine starvation leads to downregulation of the T cell receptor CD3ζ chain, impairing T cell receptor signaling and proliferation (54, 55). MDSCs also produce inducible nitric oxide synthase (iNOS), generating high levels of nitric oxide (NO). At elevated concentrations, NO induces T cell apoptosis, inhibits T cell proliferation and cytokine secretion, and disrupts NK cell cytotoxicity (54, 55). Elevated levels of ARG1 and iNOS activity in the EC-TME correlate strongly with disease progression and poor prognosis, underscoring their functional importance (56). MDSCs can also produce ROS, which further damages T cells and contributes to T cell anergy (57).

Through these multifaceted mechanisms, MDSCs establish a robust immunosuppressive network in EC, enabling tumor evasion and progression (58).

### 4.2 Therapy resistance mediated by CAFs

CAFs contribute significantly to EC progression and treatment failure through several key mechanisms (23, 59).

#### 4.2.1 Metabolic protection and chemoresistance

CAFs protect EC tumor cells from chemotherapy-induced cytotoxicity. They achieve this partly by secreting high levels of glutathione (GSH), a major cellular antioxidant. GSH neutralizes reactive oxygen species (ROS) generated by chemotherapeutic agents like platinum drugs, reducing oxidative stress and apoptosis in tumor cells (60). Furthermore, GSH can directly conjugate with platinum drugs (e.g., cisplatin), forming less reactive and more easily excreted complexes, effectively reducing the intracellular concentration of active drug (61). CAFs may also modulate the availability of other metabolites, like cysteine, which can influence drug uptake and detoxification pathways in tumor cells.

#### 4.2.2 Targetable pro-tumorigenic signaling

CAFs express high levels of fibroblast activation protein (FAP) and secrete substantial amounts of IL-6. FAP, a cell surface serine protease, is involved in ECM remodeling and promotes tumor growth and immune evasion. IL-6, as mentioned previously, stimulates tumor cell proliferation and survival via JAK/STAT3 signaling and contributes to immunosuppression. Preclinical studies in EC models suggest that targeting FAP (e.g., with inhibitory antibodies or small molecules) or blocking IL-6 signaling can inhibit CAF activity, reduce tumor growth, and sensitize tumors to chemotherapy (62, 63).

#### 4.2.3 ECM remodeling and physical barrier

CAFs are the primary architects of the desmoplastic reaction in EC, characterized by excessive deposition and cross-linking of collagen and other ECM proteins. This leads to increased stromal stiffness (64). Increased matrix stiffness physically impeders drug diffusion into the tumor parenchyma, reducing drug delivery to cancer cells. Moreover, stiffness activates mechanosensitive signaling pathways (e.g., YAP/TAZ, FAK/Src) in both tumor cells and CAFs themselves, promoting tumor cell survival, proliferation, stemness, EMT, and resistance to therapy (65). CAF-derived EVs can also carry enzymes like LOX that cross-link collagen, further increasing stiffness and promoting metastatic behavior (66).

#### 4.2.4 Paracrine signaling and survival cues

Beyond metabolites and ECM, CAFs secrete a wide array of growth factors (HGF, FGF, IGF), cytokines (IL-6, IL-8, CXCL12), and exosomes that deliver pro-survival signals directly to EC tumor cells. These signals activate PI3K/Akt, MAPK, and JAK/STAT pathways, counteracting the pro-apoptotic effects of chemotherapy and targeted therapies (67).

The profound impact of CAFs on chemoresistance and tumor progression highlights them as essential targets for improving EC treatment outcomes (68).

## 5 Therapeutic strategies aimed at the tumor microenvironment

The complexity of the EC-TME necessitates multi-faceted therapeutic approaches. Strategies targeting key immunosuppressive cells and pathways are under active development.

### 5.1 Immune checkpoint inhibitors

Immune checkpoint inhibitors (ICIs) have emerged as a critical therapeutic modality for various malignancies, demonstrating particularly significant efficacy in endometrial cancer (EC). PD-1/PD-L1 inhibitors restore T-cell anti-tumor activity by blocking the interaction between PD-1 and its ligand PD-L1 (69). The efficacy of PD-1/PD-L1 inhibitors in EC is strongly linked to the molecular subtype. Patients with mismatch repair-deficient (dMMR) or microsatellite instability-high (MSI-H) tumors, which typically have a higher tumor mutational burden (TMB) and greater immune cell infiltration, exhibit significantly higher objective response rates (ORR: 40-60%) compared to those with mismatch repair-proficient (pMMR) or microsatellite-stable (MSS) tumors, where response rates are generally low (70). This highlights the critical need for biomarker-driven patient selection.

Combination strategies are essential to overcome resistance in MSS tumors and enhance efficacy overall. Combining ICIs with agents targeting other TME components is a major focus. For example, combining ICIs with therapies that deplete or inhibit MDSCs (e.g., PDE5 inhibitors, ARG1/iNOS inhibitors, CXCR2 antagonists) has shown promise in preclinical EC models and early clinical trials, demonstrating enhanced T cell activation and improved anti-tumor responses (71, 72). This approach aims to alleviate the immunosuppressive brake applied by MDSCs, allowing unleashed T cells to effectively attack the tumor (73).

### 5.2 Rational combination therapies

Given the interconnected and redundant immunosuppressive pathways within the EC-TME, rationally designed combination therapies targeting multiple nodes simultaneously offer the greatest potential for success.

#### 5.2.1 ICI + anti-angiogenic agents

Combining ICIs with anti-angiogenic drugs (e.g., Bevacizumab - anti-VEGF antibody, Tyrosine Kinase Inhibitors - TKIs) addresses two key aspects. Anti-angiogenics normalize the aberrant tumor vasculature, reducing hypoxia, improving drug delivery, and facilitating enhanced T cell infiltration into the tumor. VEGF itself has immunosuppressive effects, inhibiting dendritic cell maturation and promoting Treg and MDSC accumulation. Blocking VEGF can thus reverse this immunosuppression and synergize with ICIs. Clinical trials in EC (e.g., NCT03526432) are evaluating such combinations (70).

#### 5.2.2 Targeting key immunosuppressive cells

TAMs: Strategies include CSF-1R inhibitors to block M2 TAM recruitment/survival and TLR agonists (e.g., TLR4, TLR9) to repolarize TAMs towards an M1 state (74, 75).

MDSCs: Pharmacological inhibitors targeting MDSC development (e.g., all-trans retinoic acid - ATRA), function (e.g., ARG1/iNOS inhibitors), or recruitment (e.g., CXCR1/2 inhibitors) are being explored. Combining these with ICIs aims to relieve MDSC-mediated suppression of T cells (72, 76).

CAFs: Approaches include neutralizing key CAF-derived factors (e.g., anti-IL-6, anti-TGF-β antibodies), inhibiting FAP enzymatic activity, or targeting specific CAF subpopulations identified via single-cell analysis. The goal is to disrupt CAF-tumor cell crosstalk, reduce ECM stiffness, and reverse chemoresistance (25).

#### 5.2.3 Disrupting EV-mediated communication

Strategies focus on inhibiting EV biogenesis, release, or uptake (e.g., using neutral sphingomyelinase inhibitors, heparin derivatives, or specific receptor blockers) to interrupt the transfer of pro-tumorigenic and immunosuppressive cargoes between cells in the EC-TME (77, 78).

These multi-targeted combination approaches represent a paradigm shift, aiming to systematically remodel the immunosuppressive EC-TME into a state conducive to effective anti-tumor immunity.

### 5.3 Extracellular vesicle based nanodelivery systems

Exploiting the inherent properties of EVs offers a novel and promising therapeutic avenue (79). EVs possess natural biocompatibility, low immunogenicity, and the intrinsic ability to cross biological barriers, making them attractive candidates as drug delivery vehicles. They can be engineered to encapsulate various therapeutic agents, including small molecule chemotherapeutics, siRNAs, miRNAs, and proteins (80). For EC therapy, engineered EVs can be designed to specifically target TME components. For instance, EVs loaded with siRNA targeting key immunosuppressive genes (e.g., Arg1, Pd-l1, Il10) in TAMs or MDSCs, and decorated with surface ligands that bind receptors enriched on these cells, can deliver their cargo directly to the intended targets, silencing gene expression and reversing immunosuppression (81). Similarly, EVs loaded with chemotherapeutic agents can be targeted to EC tumor cells or CAFs, potentially overcoming drug resistance mechanisms associated with poor tumor penetration or efflux pumps (82). This EV-based nanodelivery strategy holds the potential to significantly enhance therapeutic efficacy while minimizing off-target systemic side effects, representing a cutting-edge approach for EC treatment.

## 6 Conclusion and future perspectives

The tumor immune microenvironment of endometrial carcinoma is a dynamic and highly heterogeneous ecosystem, playing a central and complex role in disease progression, metastasis, and response to therapy. Key cellular components, including immunosuppressive TAMs, MDSCs, and CAFs, alongside non-cellular factors like EVs and the remodeled ECM, engage in intricate cross-talk to establish a profoundly immunosuppressive milieu. This environment facilitates immune evasion and confers resistance to chemotherapy, radiotherapy, and even immunotherapy. While ICIs have shown remarkable success in the dMMR/MSI-H subset of EC patients, the majority with pMMR/MSS tumors derive limited benefit, underscoring the urgent need for novel therapeutic strategies.

### 6.1 Future research directions and clinical translation

Decoding Heterogeneity with Single-Cell Technologies: The application of single-cell RNA sequencing, spatial transcriptomics, and proteomics to EC patient samples is paramount. These technologies will resolve the true diversity of cell states within the TME (distinct TAM, MDSC, CAF subpopulations, exhausted T cell subsets), elucidate their spatial organization, define specific EC-TME signatures, and identify novel cell-type-specific therapeutic vulnerabilities and biomarkers predictive of treatment response.

### 6.2 Advanced biomarker integration

Moving beyond PD-L1 IHC and MSI status, future clinical trials must systematically incorporate and validate multiplexed biomarker panels. This includes assessing tumor mutational burden (TMB), specific immune gene expression signatures (e.g., interferon-gamma), the composition and functional state of the TME (e.g., ratios of CD8+ T cells to Tregs/MDSCs, M1/M2 TAM balance), and circulating biomarkers (e.g., EV cargo, cytokine levels). Such integration is essential for refining patient stratification and enabling truly personalized therapy.

### 6.3 Rational multi-target combination therapies

Overcoming the redundancy and complexity of the EC-TME will require intelligent combination strategies. Future efforts should focus on rationally combining ICIs with agents targeting the dominant immunosuppressive mechanisms in specific EC molecular subtypes or TME contexts (e.g., ICI + TAM modulator + MDSC inhibitor; ICI + CAF-targeting agent + anti-angiogenic). Preclinical models, including patient-derived organoids and orthotopic models incorporating human immune cells, are crucial for prioritizing the most promising combinations.

### 6.4 Leveraging EVs for therapy and diagnosis

Research into EV biology in EC must expand. This includes fully characterizing the EV cargo (miRNA, protein, lipid) specific to different cell types within the EC-TME and different disease states, understanding their precise roles in mediating communication, and developing robust methods for EV isolation and engineering. EV-based therapeutics (as delivery vehicles) and diagnostics (liquid biopsies) hold immense potential for improving EC management.

### 6.5 Innovative clinical trial design

Future clinical trials need to be biomarker-driven from the outset. Adaptive trial designs (e.g., basket, umbrella trials) that allow for patient selection based on molecular/TME profiling and enable evaluation of multiple targeted combinations within a single framework are essential. Incorporating deep correlative science, including serial biopsies and multi-omics analyses, will provide critical insights into mechanisms of response and resistance.

In conclusion, advancing the treatment of endometrial cancer demands a holistic understanding of the intricate and patient-specific interactions within the TME. By leveraging cutting-edge technologies to dissect TME complexity, developing sophisticated multi-targeted therapeutic strategies, and implementing biomarker-guided clinical trials, we can move towards more effective and personalized therapies, ultimately improving survival and quality of life for EC patients. Collaborative efforts across basic science, translational research, and clinical oncology are key to unlocking the potential of TME-targeted therapies in endometrial cancer.
