# Correction: The immune microenvironment in endometrial carcinoma: mechanisms and therapeutic targeting

**DOI:** 10.3389/fimmu.2026.1785718

**Published:** 2026-01-27

**Authors:** Yilin Wang, Nana Liu, Xiangcui Guo, Ruobing Han, Jin Bai, Jiateng Zhong, Qianqing Wang

**Affiliations:** 1 Department of Gynecology , Xinxiang Central Hospital, Xinxiang, China; 2 Xinxiang Medical University, Xinxiang, China; 3 The Fourth Clinical College, Xinxiang Medical University, Xinxiang, China; 4 Henan Medical Key Laboratory for Immunology and Targeted Therapy in Gynecological Oncology, Xinxiang, China; 5 Xuzhou Medical University, Xuzhou, China

**Keywords:** endometrial carcinoma, tumor immune microenvironment, immune evasion, treatment resistance, targeted therapy, immunotherapy

Author Qianqing Wang was erroneously assigned to the original affiliation 1, Xinxiang Medical University, Xinxiang, China. This affiliation has now been removed for author Qianqing Wang. Affiliation Department of Gynecology, Xinxiang Central Hospital, Xinxiang, China, was also omitted for author Qianqing Wang. This affiliation has now been added as affiliation the new affiliation 1 and the affiliations have been reordered.

The corrected author list and affiliations are now:

Yilin Wang2,3, Nana Liu2,3, Xiangcui Guo1,3,4, Ruobing Han2,3, Jin Bai5, Jiateng Zhong2 and Qianqing Wang1,3,4*

1.Department of Gynecology , Xinxiang Central Hospital, Xinxiang, China

2.Xinxiang Medical University, Xinxiang, China

3.The Fourth Clinical College, Xinxiang Medical University, Xinxiang , China

4.Henan Medical Key Laboratory for Immunology and Targeted Therapy in Gynecological Oncology, Xinxiang , China

5. Xuzhou Medical University, Xuzhou, China.

The original version of this article has been updated.

